# Association Between Self-Reported Household Practices and Body Mass Index of US Children and Adolescents, 2005

**DOI:** 10.5888/pcd9.110149

**Published:** 2012-12-13

**Authors:** Louise C. Mâsse, Heidi M. Blanck, Maria Valente, Audie A. Atienza, Tanya Agurs-Collins, Deanne Weber, Amy L. Yaroch

**Affiliations:** **Author Affiliations:** Heidi M. Blanck, Centers for Disease Control and Prevention, Atlanta, Georgia; Maria Valente, University of British Columbia, Vancouver, British Columbia, Canada; Audie A. Atienza, Tanya Agurs-Collins, National Cancer Institute, Bethesda, Maryland; Deanne Weber, Porter Novelli, Washington, DC; Amy L. Yaroch, Gretchen Swanson Center for Nutrition, Omaha, Nebraska.

## Abstract

**Introduction:**

Parents can set household practices that influence children’s behaviors. The objective of this study was to determine whether children (children and adolescents aged 9–18 y) who live in a household that has healthful practices related to behaviors associated with obesity have a lower body mass index (BMI).

**Methods:**

We analyzed data from the 2005 Styles mail panel survey (N = 1,685 parents and children). We used multiple logistic regression to assess associations between 4 household practices and 3 children’s behaviors: watching television, participating in vigorous physical activity, and purchasing sodas and snacks at school.

**Results:**

Children watched more television if they had a television in their bedrooms, were less active as a family, and had no junk food restrictions at home. Children in less active families participated in about half as much VPA as children in more active families. Children purchased more sodas and snacks at school if they had a television in their bedrooms and their family consumed more meals at fast-food restaurants. Children whose families were less active were more likely to have a self-reported BMI at or above the 85th percentile. In addition, children who watched more television were more likely to have a self-reported BMI at or above the 85th percentile.

**Conclusion:**

Household practices were associated with children’s behaviors and self-reported BMI. A household profile that includes being active as a family may counteract the increase in childhood obesity.

## Introduction

The prevalence of obesity has increased dramatically in recent decades among American children (1). The household is recognized as a setting for intervention because it is an environment where children can develop obesogenic behaviors (2). Parents can set household practices that influence children’s weight, including limiting sedentary activities such as television viewing, emphasizing an active lifestyle, and promoting healthful eating (3–10). For example, children whose parents allow them to have televisions in their bedrooms watch more television and have a higher body mass index (BMI) (3,4,11,12). Parental practices can also influence levels of physical activity among children because active parents tend to have children who are more active (7,10). Furthermore, parents can influence the eating behaviors of their children because parents are generally in charge of purchasing food and preparing meals for the family. Limiting access to and availability of less healthful foods (eg, sodas, high-fat snacks) at home has been associated with lower BMI, higher consumption of fruits and vegetables, and decreased consumption of foods with low dietary quality (13–15). Conversely, children in homes where family meals are regularly purchased from fast-food restaurants are less likely to consume vegetables or milk with meals and more likely to have a high BMI (16–18).

Previous studies have not compared household practices with children’s weight-related behaviors. The main objective of this study was to assess whether children and adolescents who live in households that have healthful practices watch less television, engage in more physical activity, and purchase fewer sodas and snacks at school. A secondary aim of this study was to examine whether healthful household practices and children’s behaviors were associated with lower self-reported BMI.

## Methods

We conducted a secondary data analysis of the 2005 Styles mail panel survey (ConsumerStyles, HealthStyles, and YouthStyles) because it included a household module. Styles is a proprietary consumer and health behavior database developed by Porter Novelli with data collected by Synovate, Inc. In 2005, the Centers for Disease Control and Prevention and the National Cancer Institute purchased the rights to analyze the de-identified data. Because Porter Novelli/Synovate, Inc, funds and conducts data collection, approval of the Office of Management and Budget and the institutional review boards is not necessary. Porter Novelli/Synovate, Inc, adheres to all professional standards and codes of conduct set by the Council of American Survey Research Organizations (www.casro.org/pdfs/10CodeOfStandards.pdf).

### Participants

Panel members were first surveyed with the ConsumerStyles survey. The ConsumerStyles survey, conducted May through June 2005, was sent to a stratified random sample of 20,000 adult panel members (N = approximately 450,000 adults aged 18 or older), which was balanced to help create a nationally representative sample. Low income and minority subgroups (blacks and Hispanics) and households with children were oversampled. The response rates were 65% for the main sample, 59% for the low-income and minority sample, and 62% for households with children.

From July through August 2005, approximately half (n = 6,209) of the panel members who completed the ConsumerStyles survey were followed up with the HealthStyles survey for households without children (n = 3,692) or with both the HealthStyles and YouthStyles surveys (n = 2,517) for households with children. Of the 6,209 panel members who received the follow-up surveys, 4,943 (80%) completed them. The HealthStyles survey was completed by an adult or parent in the household. The YouthStyles survey, mailed in conjunction with the HealthStyles survey, was completed by 1 child in the household. The YouthStyles survey targeted children aged 9 to 18 years and provided linked data between parents and their children. Of the 2,517 households with children that received both the HealthStyles and YouthStyles surveys, 1,685 (67%) completed both surveys. Participants who responded to these surveys were nominally compensated for their participation ($1-5).

Of the 1,685 participants who completed both the HealthStyles and YouthStyles surveys, we excluded those who had missing demographic data (n = 30) and those with missing responses to the questions that asked whether children have a television in their bedroom (n = 210), they are active as a family (n = 18), they allow junk food consumption (n = 5), or they go to fast-food restaurants as a family (n = 10). For the analyses that examined whether the household practices were associated with child or adolescent television watching, physical activity, and dietary behaviors, we excluded participants with missing responses to these questions (n = 232), resulting in an effective sample size of 1,190. The analyses that included BMI percentiles had an effective sample size of 1,050 (missing BMI percentiles, n = 140). The panel members included in the analyses, compared with those excluded for the reasons cited above, had a significantly higher weighted proportion of children aged 9 to 12 (46.8% vs 38.8%), whites (71.7% vs 59.5%), families with higher education (34.6% vs 23.7%), higher income families (39.0% vs 28.0%), more parents aged 35 to 44 (46.2% vs 41.6%), and a higher proportion of families with married parents (84.5% vs 76.3%).

### Measures

Parents self-reported their age, sex, education, race/ethnicity, marital status, and annual household income. Household practices were assessed with 4 questions. Parents were asked, “Does your child have a television in his/her room?” (yes/no). Using a 5-point response format (strongly agree, agree, neutral, disagree, and strongly disagree), we asked parents to what extent they agreed with the following statements: 1) “I don’t allow my children to eat junk food,” and 2) “We are active as a family.” Responses were recoded into 3 categories (agree/neutral/disagree) for the analyses. Family visits to fast-food restaurants were assessed with the question, “How many days per week do you take your children out to eat at fast-food restaurants, such as . . . ?” Answers ranged from 0 to 7 days.

Age and sex of children and adolescents were reported by their parents. Children self-reported their height and weight, which were used to calculate BMI percentile categories; children with a BMI at or above the 85th percentile and less than the 95th percentile were classified as overweight, and those with a BMI at or above the 95th percentile were classified as obese (19–22). Television-watching time was assessed with 2 questions: “After school, on an average weekday (Monday to Friday) during the school year, how many hours a day do you spend watching television?” and “On an average weekend (both Saturday and Sunday) during the school year, how many hours a weekend do you spend watching television?” For both questions, the responses were 0 hours, less than 1, 1, 2, 3, 4, and 5 or more hours. Eating behavior at school was assessed with the following question: “How many days in a typical week did you buy sodas or other snack foods such as chips, chocolate bars, or cookies from vending machines at school?” Answers ranged from 0 to 5 days. Vigorous physical activity (VPA) was assessed with 1 question: “On how many days of the past 7 days did you exercise or participate in physical activity for at least 20 minutes that made you sweat or breathe hard?” Examples were provided and answers ranged from 0 to 7 days. For the analyses, those who were active at least 3 days were classified as meeting the VPA recommendation, those who were active 1 to 2 days were classified as doing some VPA, and those who reported 0 days were classified as doing no VPA. The television watching and VPA questions were selected from the Youth Risk Behavior Surveillance System (YRBSS) survey; the television questions were modified to ask about television watching behaviors on weekday and weekend days instead of just focusing on an average school day (23). The validity of these questions has been assessed previously with accelerometers (*r* = 0.36 for the VPA question, *r* = 0.37 for television watching on weekday, and *r *= 0.47 for television watching on the weekend) (24,25).

### Statistical analysis

Stata version 11.0 (StataCorp LP, College Station, Texas) was used for all the analyses. All analyses were weighted so that the results would be reflective of US families in terms of race/ethnicity, sex, household size, and income matched to the US Census 2004.

Three multivariate polytomous logistic regressions were performed to examine the associations between household practices and the behaviors of children and adolescents (ie, television watching, participation in physical activity, and purchasing sodas and snacks at school). All the household practices were entered as independent variables because parental practices may be inconsistent across areas (eg, families that provide their child with a television in his or her bedroom may or may not have rules regarding access to less healthful foods). A multivariate polytomous logistic regression was performed to examine associations with self-reported BMI percentile categories; household practices and the behaviors of children and adolescents were entered as independent variables.

For all the analyses, sex and age of the child, race/ethnicity of family, and parent education were entered as covariates. Income was also considered as a covariate; however, education had a stronger association and when both variables were entered, income did not remain significant. A Bonferroni correction was used to control for family-wise error rate. Significance was set at *P* < .01 (.05/4 hypotheses = .013).

## Results

### Descriptive data

The sample comprised predominantly white families (72%) (Table 1). Less than 50% of children reported 14 or less hours of television watching per week; 72% reported engaging in VPA at least 3 times a week, and 43% purchased sodas and snacks at schools at least once a week. In addition, approximately one-third of parents reported having a college degree, 62% of the children and adolescents had a television in their bedroom, 21% of the families were not active as a family, few families reported having junk food restrictions (13%), and more than 75% of the families went to fast-food restaurants at least once a week.

**Table 1:** Characteristics, Behaviors, and Household Practices, by Body Mass Index Percentile Categories, of US Children and Adolescents (N = 1,050), HealthStyles and YouthStyles Survey, 2005

Characteristics and Household Practices	Total^a^ (%)	BMI^b^		

		<85th Percentile (%)	≥85th and <95th Percentile (%)	≥95th Percentile (%)
**Sex^c^**				
Male	49.2	60.8	18.1	21.1
Female	50.8	68.0	15.7	16.3
**Age, y^c^**				
9–12	46.8	56.9	20.8	22.3
13–15	29.5	67.7	12.8	19.6
16–18	23.7	75.3	14.3	10.4
**Parental education^c^**				
College graduate	34.6	70.8	14.2	15.0
Some college	41.0	64.3	17.4	18.3
High school diploma or less	24.5	55.6	19.9	24.5
**Race/ethnicity^c^**				
White	71.7	67.5	16.9	15.6
Black	8.7	51.6	21.3	27.0
Hispanic	13.9	56.7	15.8	27.5
All other groups	5.7	64.8	12.6	22.6
**Television watching^b^**				
≤14 h/wk	46.9	69.0	16.4	14.7
>14–<21 h/wk	30.7	62.8	19.0	18.2
≥21 h/wk	22.5	57.3	15.2	27.6
**Vigorous physical activity (VPA)^b^**				
Meets recommendations^d^	72.2	65.6	16.7	17.7
Some	18.1	59.9	18.9	21.2
None	9.7	64.0	14.7	21.4
**Purchases sodas and snacks^b^**				
0 d/wk	57.3	65.7	16.1	18.2
1–2 d/wk	27.8	60.7	18.4	21.0
3–5 d/wk	14.9	66.8	17.0	16.2
**Television in bedroom^c^**				
No	38.5	70.4	14.6	15.0
Yes	61.5	60.7	18.3	21.0
**Active as a family^c^**				
Agree/strongly agree	41.1	68.7	17.2	14.1
Neutral	38.0	65.1	15.4	19.5
Disagree/strongly disagree	21.0	54.9	19.1	26.1
**Junk food restriction^c^**				
Agree/strongly agree	12.8	61.0	18.3	20.7
Neutral	38.9	63.4	16.1	20.5
Disagree/strongly disagree	48.3	66.2	17.2	16.6
**Eats at a fast-food restaurant^c^**				
0 d/wk	24.1	71.1	14.3	14.7
1 d/wk	46.0	63.8	18.3	17.9
≥2 d/wk	29.9	60.1	16.8	23.1

Abbreviations: BMI, body mass index.

a Percentages may not add to 100% because of rounding.

b YouthStyles variable; calculated on the basis of self-reported data from the child.

c HealthStyles variable; self-reported data from the parent.

d Defined as doing at least 20 minutes of VPA on at least 3 days of the week.

### Associations with household practices

Black children and adolescents were more likely to watch television than white children and adolescents (OR, 1.90) (Table 2). In addition, children and adolescents watched more television if they had a television in their bedroom (OR = 1.58), were less active as a family (OR = 1.84), and had fewer junk food restrictions at home (OR = 1.83).

**Table 2:** Association of Household Practices With Behaviors of US Children and Adolescents (N = 1,190), HealthStyles and YouthStyles Survey, 2005

Characteristics and Household Practices		Television Watching^a^^,^ b	Vigorous Physical Activity^a^^,^ c	Purchasing Sodas or Snacks^a^^,^ d

		OR (95% CI)	OR (95% CI)	OR (95% CI)
**Sex^e^**				
Male		1 [Reference]		
Female		0.80 (0.63–1.01)	0.70 (0.53–0.92)	0.98 (0.77–1.26)
**Age, y^e^**				
9–12		1 [Reference]		
13–15		1.14 (0.87–1.49)	1.17 (0.84–1.63)	3.50 (2.61–4.69)^f^
16–18		1.21 (0.89–1.64)	0.65 (0.46–0.91)	4.07 (2.96–5.61)^f^
**Parental education^e^**				
College graduate		1 [Reference]		
Some college		1.36 (1.04–1.79)	0.94 (0.66–1.31)	1.24 (0.92–1.67)
High school diploma or less		1.33 (0.98-1.80)	0.76 (0.53-1.10)	1.18 (0.84-1.65)
**Race/ethnicity^e^**				
White		1 [Reference]		
Black		1.90 (1.34–2.69)^f^	1.30 (0.83–2.04)	2.01 (1.35–3.00)^f^
Hispanic		1.22 (0.85–1.75)	0.67 (0.47–0.95)	1.67 (1.21–2.32)^f^
All other groups		0.74 (0.48–1.14)	1.21 (0.66–2.22)	0.91 (0.56–1.49)
**Television in bedroom^e^**				
No	1 [Reference]			
Yes		1.58 (1.22–2.05)^f^	0.93 (0.69–1.24)	1.49 (1.13–1.96)^f^
**Active as a family^e^**				
Agree		1 [Reference]		
Neutral		1.28 (0.97–1.68)	0.51 (0.37–0.70)^f^	1.04 (0.78–1.38)
Disagree		1.84 (1.34–2.52)^f^	0.35 (0.25–0.51)^f^	1.20 (0.86–1.69)
**Junk food restriction^e^**				
Agree		1 [Reference]		
Neutral		1.56 (1.06–2.32)	0.92 (0.58–1.48)	1.38 (0.92–2.07)
Disagree		1.83 (1.24–2.70)^f^	0.85 (0.54–1.34)	1.20 (0.81–1.80)
**Eats at a fast food restaurant^e^**				
0 d/wk		1 [Reference]		
1 d/wk		1.03 (0.77–1.37)	1.34 (0.94–1.91)	1.62 (1.18–2.24)^f^
≥2 d/wk		1.25 (0.91–1.71)	1.08 (0.74–1.58)	1.67 (1.19–2.36)^f^

Abbreviations: OR, odds ratio; CI, confidence interval.

a YouthStyles variable: self-reported data from the child.

b Overall model fit is *χ* ^2^ (*df *= 15) = 6.47; *P* < .001.

c Overall model fit is *χ* ^2^ (*df* = 15) = 4.85; *P* < .001.

d Overall model fit is *χ* ^2^ (*df* = 15) = 11.55; *P* < .001.

e HealthStyles variable; self-reported data from the parent.

f Significant at *P* < .01.

None of the demographic variables were associated with VPA at *P* < .01 (Table 2). Children in less active families participated in about half as much VPA as children in more active families (OR = 0.51 for somewhat active families, and OR = 0.35 for inactive families).

The age of the child was related to purchasing sodas and snacks at school (Table 2). Children aged 13 to 15 were more likely to report purchasing sodas and snacks at school than children aged 9 to 12 (OR = 3.50). In addition, children aged 16 to 18 were more likely to report purchasing sodas and snacks at school than children aged 9 to 12 (OR = 4.07). Race/ethnicity was also related to purchasing behaviors at school. Black (OR = 2.01) and Hispanic children and adolescents (OR = 1.67) were more likely than white children and adolescents to report purchasing sodas and snacks at school. Having a television in the bedroom and eating at fast-food restaurants “as a family” were also related to purchasing behaviors at school. Children and adolescents with a television in their bedroom (OR = 1.49) were more likely to purchase sodas and snacks at school than those without a television. Children whose families went to fast-food restaurants at least once a week were more likely to report purchasing sodas and snacks at school than those who did not go to fast-food restaurants (OR = 1.62 for visiting a fast-food restaurant 1 day per week and OR = 1.67 for 2 or more days per week).

### Associations with self-reported BMI percentile categories

In Model 1 (covariate and behavior variables model), television watching was the only behavior associated with self-reported BMI percentile categories among children and adolescents (Table 3). Those who reported watching more than 21 hours of television per week (OR = 1.72) were more likely to have higher self-reported BMI percentile categories than those who reported watching 14 hours or less of television per week. In Model 2 (covariate and household practice model), being active as a family was the only household practice that was significantly associated with self-reported BMI percentiles. Children and adolescents whose families were less active were more likely to have higher self-reported BMI percentile categories (OR = 2.21) than those who had active families. When the 3 behaviors and the 4 household practices were entered into the model (Model 3), television watching (OR = 1.64) and being active as a family (OR = 2.05) remained significant.

**Table 3:** Association of Behaviors and Household Practices With Self-Reported Body Mass Index (BMI) Percentile Categories, US Children and Adolescents (N = 1,050), HealthStyles and YouthStyles Survey, 2005

Characteristics and Household Practices	Model 1^a^	Model 2^b^	Model 3^c^

	OR (95% CI)	OR (95% CI)	OR (95% CI)
**Sex^d^**			
Male	1 [Reference]		
Female	0.72 (0.54–0.95)	0.72 (0.54–0.95)	0.71 (0.53–0.95)
**Age, y^d^**			
9–12	1 [Reference]		
13–15	0.63 (0.45–0.89)^e^	0.64 (0.46–0.89)^e^	0.62 (0.44–0.87)^e^
16–18	0.38 (0.26–0.54)^e^	0.39 (0.27–0.55)^e^	0.36 (0.25–0.52)^e^
**Parental education^d^**			
College graduate	1 [Reference]		
Ηigh school diploma or less	1.74 (1.22–2.48)^e^	1.68 (1.18–2.38)^e^	1.61 (1.13–2.31)^e^
Some college	1.19 (0.84–1.67)	1.15 (0.83–1.61)	1.11 (0.79–1.55)
**Race/ethnicity^d^**			
White	1 [Reference]		
Black	1.77 (1.15–2.73)^e^	1.72 (1.12–2.64)	1.66 (1.07–2.56)
Hispanic	1.47 (0.99–2.18)	1.41 (0.96–2.06)	1.34 (0.90–1.97)
All other groups	1.27 (0.71–2.29)	1.03 (0.56–1.88)	1.08 (0.57–2.02)
**Television watching^f^**			
≤14 h/wk	1 [Reference]		
>14–<21 h/wk	1.24 (0.90–1.70)	NA	1.14 (0.82–1.58)
≥21 h/wk	1.72 (1.19–2.48)^e^	NA	1.64 (1.12–2.39)^e^
**Vigorous physical activity (VPA)^f^**			
Meets recommendation^g^	1 [Reference]		
Some	1.21 (0.75–1.95)	NA	1.08 (0.67–1.76)
No	1.36 (0.96–1.92)	NA	1.31 (0.92–1.85)
**Purchasing sodas and snacks^f^**			
0 d/wk	1 [Reference]		
1–2 d/wk	1.35 (0.97–1.88)	NA	1.27 (0.91–1.78)
3–5 d/wk	0.96 (0.62–1.48)	NA	0.90 (0.58–1.40)
**Television in bedroom^d^**			
No	1 [Reference]		
Yes	NA	1.39 (1.02–1.89)	1.35 (0.98–1.87)
**Active as a family^d^**			
Agree	1 [Reference]		
Neutral	NA	1.32 (0.96–1.82)	1.28 (0.92–1.77)
Disagree	NA	2.21 (1.52–3.21)^e^	2.05 (1.42–2.98)^e^
**Junk food restriction^d^**			
Agree	1 [Reference]		
Neutral	NA	0.95 (0.63–1.43)	0.93 (0.62–1.40)
Disagree	NA	0.70 (0.46–1.07)	0.66 (0.43–1.01)
**Eat at fast-food restaurant^d^**			
0 d/wk	1 [Reference]		
1 d/wk	NA	1.17 (0.82–1.66)	1.18 (0.83–1.69)
≥2 d/wk	NA	1.31 (0.89–1.94)	1.30 (0.88–1.92)

Abbreviations: OR, odds ratio; CI, confidence interval; NA, not applicable (variable is not included in the model).

a Model 1 includes all the covariates and behavior variables (*χ* ^2^ [*df* = 14] = 5.58; *P* < .001).

b Model 2 includes all the covariates and household variables (*χ* ^2^ [*df* = 15] = 5.11; *P* < .001).

c Model 3 includes all the covariates and all the variables (*χ* ^2^ [*df* = 21] = 4.38; *P* < .001).

d HealthStyles variable; self-reported data from the parent.

e Significant at *P* < .01.

f YouthStyles variable; self-reported data from the child.

g Defined as doing at least 20 minutes of VPA on at least 3 days of the week.

## Discussion

Television watching, participation in VPA, and purchasing of sodas and snacks at school were all found to be associated with household practices at home. Our findings parallel those of other studies that reported children who have a television in their bedroom watched more television (3,4,11,12,26) and that children in active families performed more VPA (7,10).

To our knowledge, no other studies have simultaneously examined whether multiple household practices are associated with children’s behavior outside the home. We found that children whose families often go out to fast-food restaurants are more likely to purchase sodas and snacks at school. We anticipated this finding because children who often go out to fast-food restaurants as a family have generally less healthful diets (16–18). However, no association was found with having junk food restrictions at home and purchasing behaviors at school. It appears that when children are faced with many food choices outside the home, having restrictive food practices at home does not track into other settings; such an approach may not allow children to practice making healthful choices outside the home. Future studies might be able to further elucidate this lack of association by testing this explanation.

A cluster of household practices were associated with television watching, but this pattern was not observed for participation in VPA. We found that children who have a television in their bedrooms watched more television; however, those who live in an active family and have junk food restrictions at home watched less television. In contrast, participation in VPA was associated with only 1 household practice: being active as a family. Finding a different set of household practices to be associated with these behaviors highlights that television watching is likely influenced by setting restrictions in the household (3,4,11,12), whereas engaging in physical activity is likely influenced by modeling the behavior and by encouraging and supporting participation (7,10). This may explain why we found that other restrictions, such as junk food restrictions, were associated with television watching and not with participation in VPA. Because our study did not fully address the spectrum of household practices associated with television watching and participation in VPA, household practices related to these behaviors should be examined further.

Another goal of this study was to examine associations with self-reported BMI. In our study, television watching was the only behavior significantly associated with self-reported BMI, whereas participation in VPA and purchasing of sodas and snacks at school were not, although the former approached significance (*P* = .054). Our study did not assess dietary intake but measured a specific dietary pattern (eg, purchasing of sodas and snacks at school), which may explain this lack of association. Furthermore, other researchers have reported conflicting findings regarding the association of television watching and physical activity with BMI (27). Alternatively, the use of self-reported BMI may explain our lack of associations; self-reported BMI has been shown to be less accurate in children younger than 12 (association reported to range from .52 to .70 compared with .79 to .93 in older children) (28).

Being active as a family was the only household practice significantly associated with self-reported BMI. Being active as a family may be a surrogate measure for healthy family behaviors that include participation in physical activity as well as other behaviors such as more healthful eating and less television watching. This finding underscores the need to better understand the profiles of active families, a subject that has not been examined in depth.

Our study has limitations. The cross-sectional nature of the data limits our ability to examine causality. Although we acknowledge the potential limitations of self-report, the health behavior measures were associated with the household variables as expected and, as such, support the potential validity of these measures. Although television watching is a relevant sedentary behavior, this study did not account for all other sedentary activities in which children engage. Because the household variables were developed for this study, the validity of these measures warrants further investigation. In addition, we were unable to capture the full range of practices that may be associated with the 3 behaviors we examined and BMI. The potential effect of the school environment was not examined but is acknowledged as a limitation, given its potential influence on behaviors. Lastly, the data were collected using a consumer opinion panel, which may limit the generalizability of the findings; however, this technique yields results comparable to those of random-digit dialing (29).

Our study simultaneously examined multiple household practices in relation to 3 behaviors and self-reported BMI among children and adolescents. Our findings provide evidence that the household environment is associated with certain behaviors and suggests that being active as a family might be relevant for childhood obesity prevention.
